# Numerical investigation of the translational motion of bubbles: The comparison of capabilities of the time-resolved and the time-averaged methods

**DOI:** 10.1016/j.ultsonch.2022.106253

**Published:** 2022-12-06

**Authors:** Kálmán Klapcsik, Ferenc Hegedűs

**Affiliations:** Department of Hydrodynamic Systems, Faculty of Mechanical Engineering, Budapest University of Technology and Economics, Műegyetem rkp. 3., H-1111 Budapest, Hungary

**Keywords:** Keller-Miksis equation, Bubble dynamics, Bubble translation, Acoustic forces

## Abstract

In the present study, the accuracies of two different numerical approaches used to model the translational motion of acoustic cavitational bubble in a standing acoustic field are compared. The less accurate but less computational demanding approach is to decouple the equation of translational motion from the radial oscillation, and solve it by calculating the time-averaged forces exerted on the bubble for one acoustic cycle. The second approach is to solve the coupled ordinary differential equations directly, which provides more accurate results with higher computational effort. The investigations are carried out in the parameter space of the driving frequency, pressure amplitude and equilibrium radius. Results showed that both models are capable to reveal stable equilibrium positions; however, in the case of oscillatory solutions, the difference in terms of translational frequency may be more than three fold, and the amplitude of translational motion obtained by the time-averaged method is roughly 1.5 times higher compared to the time-resolved solution at particular sets of parameters. This observation implies that where the transient behaviour is important, the time-resolved approach is the proper choice for reliable results.

## Highlights

• In the present study, the accuracies of the time-resolved and time-averaged approaches are compared for modelling the translational motion of acoustic cavitational bubbles in a standing acoustic field.
• The investigations are carried out in the parameter space of the driving frequency, pressure amplitude and equilibrium radius by utilizing the high processing power of GPUs.
• For positionally unstable bubbles, at sets of parameters, the difference in translational frequency may be more than three and the amplitude of translational motion obtained by the time-averaged method is roughly 1.5 times higher than the time-resolved solution.
• When the transient translational motion is important, the time-resolved approach is the proper choice for reliable results.

## 1 Introduction

A keen interest in sonochemistry is to utilize ultrasound in chemical reactions. Its physical background is the acoustic cavitation. Due to the high amplitude acoustic waves, bubble clusters consisting of thousands of radially pulsating bubbles are formed in the liquid domain. If the intensity of the irradiation is sufficiently high, the bubbles expand many time larger than their equilibrium size, then violently collapse. The collapse is stopped by the dramatically increased pressure inside the bubble. Near the minimum radius, the temperature can also reach thousands of degrees of Kelvin, which induce chemical reactions in the bubble interior [1], [2], [3], [4], [5], [6], [7] and shock waves are generated [8], [9], [10], [11].

The free radicals produced via the bubble collapse are used in wastewater treatment [12], [13], [14], [15], [16], or in degradation and oxidation of pollutants [17], [18], [19]. For example, to degrade the hazardous chemical species, such as Perfluorooctanesulfonic acid (PFOS), sonochemistry seems to be the only option [20]. Some novel applications of sonochemistry are the production of hydrogen (Green fuel) [21], [22], [23] and the production of nanoalloys and nanoparticles [24], [25], [26], [27]. In addition, ultrasound can be used to enhance the synthesis of organic compounds or pharmaceuticals by using fewer toxic or toxic-free solvents [28] (Green Chemistry).

The biggest challenge in sonochemistry is to scale up the applications feasible for industrial/commercial size. The main barriers of scalability are 1) the dependence on operating parameters, 2) the high number of involved parameters, 3) the non-uniform cavitational activity, and 4) the attenuation of sound waves [29], [30], [31]. Many papers deal with the dependence on operating parameters by investigating the nonlinear nature of single bubble [32], [33], [34], [35], [36], [37], [38], [39], [40], [41], [42], [43], [44], [45], [46]. As the number of the involved parameters is large, the computing power of GPUs is utilized to investigate the wide range of parameter space [47]. To face with the challenge of non-uniform cavitational activity and the attenuation of sound waves, one has to deal with many-bubble systems. One of the key phenomena related to multi-bubble systems is the translational motion of bubbles besides the volume and shape oscillation. Nevertheless, translational motion is a less researched subject, while the spatial location of individual bubbles is of great practical importance to minimize the attenuation of sound waves caused by densely packed bubbles (acoustic shielding [48]) or decrease the volume of passive zones formed in the reactor [49]. From linear theory, it is known that bubbles with size below the resonance size are attracted by the pressure antinode, while bubbles larger than the resonance size are pushed to the pressure node in a standing acoustic field. However, in the nonlinear regime, the repulsion of bubbles from the pressure antinode can be observed. Thus; the bubble accumulation in parallel layers is observed [50], [51], [52], [53], [54]. As the bubbles behave as mechanical oscillators, wave dispersion is expected. The increasing void fraction in bubbly media changes the acoustic properties of the liquid, such as the speed of sound and the nonlinear attenuation [31], [55], [56], [57], [58]. For close bubbles, the effect of bubble–bubble interaction is not negligible. Even for a small void fraction, the increasing pressure amplitude and the bubble–bubble interaction has huge influence on the properties of the acoustic field compared to the non-bubbly liquid [59]. The numerical simulations of Louisnard showed that the acoustic energy is dissipated in thin layer of oscillating bubbles [60]. A possible option to moderate the shielding effect is to control the behaviour of the bubble cluster. Control techniques such as a simple ON/OFF control [61], [62], the application of bi-frequency driving to avoid the spatial instability [63], [64] or the optimization of the geometry [65] already exist in the literature. A possible approach is to apply acoustic manipulation methods to control the position of individual bubbles. Acoustic manipulation methods are already extensively used for solid particles [66], [28], [67]. However, their application for cavitation bubbles is not yet widespread. Exceptions exist for single bubbles [68], where bubble position control was achieved by shifting the acoustic wave field using opposite transducers with a variable phase shift difference attached at the end of the liquid column. The manipulation of bubbles can be highly challenging due to the nonlinear behaviour of the bubbles and the spatially complex acoustic field generated in a sonochemical reactor [69], [70], [71], [72]. The authors believe that the success of developing a feasible control method for real applications highly depends on numerical simulations. Due to the huge amount of involved parameters, efficient computational methods are required to exploit the capabilities of high-performance computing to investigate the dynamics in detail. For example, in a recent study, employing a six-dimensional parameter space even with a moderate resolution, the number of different parameter combinations was nearly 2 billion [47]. Besides being computationally efficient, an accurate modelling approach is required without oversimplification to obtain reliable results. Thus; the present paper aims to compare the two qualitatively different modelling approaches available in the literature, which are used to describe the translational motion of a radially pulsating bubble.

The translational motion of bubbles is governed by the acoustic radiation forces [73], [74], [75], [76], which are usually classified as *primary- and secondary Bjerknes forces*. The primary Bjerknes force describes the effect of acoustic pressure gradient on single bubbles, and it gathers the bubbles in certain areas, such as pressure nodes or antinodes in the case of a standing sound field [77], [78], [79], [78]. The bubble–bubble interaction via the emitted pressure is described by the secondary Bjerknes force that can attract or repel neighbouring bubbles [80], [81], [82]. In addition, the bubble–bubble interaction has an influence on the radial dynamics as well, which was observed numerically [83], [59], [84] and experimentally [85]. The effect of these radiation forces leads to the formation of stable bubble structures [52], [54].

To obtain the individual bubble paths in an acoustic wave field, two kinds of numerical approaches exist. One possible candidate is to describe the translational behaviour of bubbles in terms of time-averaged approach, which treats every bubble as a single particle accelerated by external forces such as acoustic radiation forces (discussed above), drag force and added mass force. These instantaneous forces exerted on the bubble by the surrounding liquid are averaged over one acoustic period (1/f). Then, the bubble positions and velocities are updated by solving the equation of motion. Although, this approach is justified for weak acoustic fields with small radial oscillation and translation motion; it is able to qualitatively capture the features of caviation clusters [50], [52], [53].

The second approach is to solve the differential equations describing the instantaneous bubble translation and oscillations. Watanabe and Kukita [86] investigated the translational motion by applying Newton’s Second Law directly to a bubble immersed in the liquid domain. However, their model does not involve the direct feedback effect of the translational motion on the radial pulsation. Doinikov [79], [87] refined the model, by using the Lagrangian formalism, which includes the direct coupling between the translational and radial motions. The interaction of two bubbles is taken into account via coupled equations of radial and translational motions. Doinikov [80] and Harkin et al. [88] derived coupling terms with an accuracy up to third order and fourth in the inverse distance, respectively. The theoretical model was refined for arbitrary distances between the bubble by taking into account the coupling terms up to tenth order in [89]. The time-resolved approach was generalized for multi-bubble systems with arbitrary spatial arrangement with coupling terms up to third order in inverse distance [90]. Note that the detailed theoretical review of the bubble translation is provided in [81].

The comparison of the numerical techniques has already been given in the literature for a limited amount of parameter sets. Reddy and Szeri [91] found that the decoupling results in an error of approximately 35% and 120% in terms of the averaged velocity in the case of strong collapse in sub-resonant driving and mild collapse in super-resonant driving. Kreftig et al. [92] investigated a broader range of driving frequencies and bubble sizes. An error of 30% in terms of translational speed obtained by the decoupling approximation relative to the translation speed obtained by the full equations was observed even for a weak acoustic field. However, the local error observed in the translational velocity does not indicate the qualitatively different translational dynamics of the bubble. Thus, the present study investigates the accuracy of both approaches numerically on a wide range of parameter combinations, which is *not limited to weak forcing*.

The numerical results provided in the present paper aid in finding a compromise between accuracy and computational effort. The computations were carried out in plane standing waves. For simplicity, the translational motion of the bubbles were restricted to one spatial dimension. The results showed that both modelling approaches provide reliable results in seeking stable equilibrium. On the contrary, in the case of transient bubble translational motions, the results showed poor agreement. This observation implies that in the case of cluster simulations, where the transient behaviour is inevitable, the time-resolved approach is the proper choice especially when position control is the main aim.

## 2 Mathematical model

The radial dynamics of the bubble is described by the Keller–Miksis equation [93]. The translational motion is modelled by two different approaches. The simplest one is to decouple the equation of motion from the differential equation describing the radial oscillation and solve it separately by considering the time-averaged forces acting on the bubble by averaging over one cycle of acoustic period. The approach of averaging and decoupling to calculate the averaged bubble path was applied to qualitatively explain structure formation of acoustic cavitation cluster [50]. In the present paper, this approach is referred as *Time-Averaged* method. The second approach is to solve the full *Time-Resolved* equation of motion coupled to the radial oscillation [78], [80], [87]. The governing equations are summarized below. Note that in the present paper the translational motion is restricted to one dimensional translation; thus, there is only one non-zero space coordinate.

### 2.1 Radial oscillations

The radial dynamics of a spherical bubble in viscous, compressible liquid is described by the Keller–Miksis equation [93] reads as

(1) $$\left(1-\frac{\overset{˙}{R}}{c_{L}}\right)R\overset{¨}{R}+\left(1-\frac{\overset{˙}{R}}{3c_{L}}\right)\frac{3}{2}{\overset{˙}{R}}^{2}=\left(1+\frac{\overset{˙}{R}}{c_{L}}+\frac{R}{c_{L}}\frac{d}{\mathrm{dt}}\right)\frac{\left(p_{L}-p(x,t)\right)}{\rho_{L}}+\frac{{\overset{˙}{x}}^{2}}{4},$$

where the extension of the last term takes into account the feedback from the translational motion (${\overset{˙}{x}}^{2}/4$). Note that this last term is omitted in the time-averaging approach. In the above equation, *R* and *x* are the instantaneous bubble radius and position; respectively. Furthermore, $\rho_{L}=998\;\mathrm{kg}/m^{3},c_{L}=1500\;m/s$, and $p_{L}$ are the liquid density, the sound speed and the pressure in the liquid at the bubble wall, respectively. Moreover, $p_{\infty }(x,t)$ denotes the far field pressure, which depends on the acoustic field, see Section 2.4. Note that the dots stand for derivatives with respect to time.

The connection between the inner and outer pressure is described by a mechanical balance of normal stresses at the bubble wall

(2) $$p_{G}+p_{V}=p_{L}+\frac{2\sigma }{R}+4\mu_{L}\frac{\overset{˙}{R}}{R},$$

where σ=0.0725N/m and $\mu_{L}=0.001\;\mathrm{Pa}\;s$ are the surface tension, and liquid dynamic viscosity, respectively. The internal pressure is the sum of the partial pressures of non-condensable gas $p_{\mathrm{Gn}}$ and vapour $p_{V}=0\;\mathrm{Pa}$ (pure gas bubble).

The gas pressure inside the bubbles is calculated according to a polytrophic relationship

(3) $$p_{G}=\left(\frac{2\sigma }{R_{0}}-p_{V}+P_{\infty }\right){\left(\frac{R_{0}}{R}\right)}^{3\gamma },$$

where γ=1.4 is the polytropic exponent (adiabatic behaviour), and $R_{0}$ is the equilibrium radius of the bubble.

### 2.2 Translation modelled by the time-resolved approach

The translational motion of the bubble is described as [78], [86]

(4) $$m_{b}\overset{¨}{x}+\frac{1}{2}\rho_{L}\frac{d}{\mathrm{dt}}\left(V\overset{˙}{x}\right)=F_{\mathrm{ex}}(x,t),$$

where $m_{b}$ is the mass of the gas and vapour inside the bubble, $F_{\mathrm{ex}}(x,t)$ is the instantaneous external force acting on the bubble and $V(t)=4\pi R^{3}/3$ is the bubble volume. After the derivative of the product is expressed, and the equation is reorganized the translational motion of the bubble is described as

(5) $$\left(m_{b}+\frac{1}{2}\rho_{L}V\right)\overset{¨}{x}+\frac{1}{2}\overset{˙}{V}\overset{˙}{x}=F_{\mathrm{ex}}(x,t)\text{.}$$

The mass of the bubble $m_{b}$ is negligible compared to the virtual mass term $1/2\rho_{L}V$; thus, after substituting the bubble volume $V=4\pi R^{3}/3$ and its time derivative $\overset{˙}{V}=4R^{2}\pi \overset{˙}{R}$ into the equation above, the equation of translational motion is written as [79], [81]

(6) $$R\overset{¨}{x}+3\overset{˙}{R}\overset{˙}{x}=\frac{3F_{\mathrm{ex}}(x,t)}{2\pi \rho_{L}R^{2}}\text{.}$$

The external force $F_{\mathrm{ex}}(x,t)$ is the sum of the instantaneous primary Bjerknes force and the viscous drag force. The primary Bjerknes force, which is induced by the acoustic pressure gradient, is calculated as

(7) $$F_{B1}=-V(t)\nabla p_{A}(x,t),$$

where $\nabla p_{A}(x,t)$ is the pressure gradient at the centre of the bubble. The drag force is obtained according to the Levich formula [94]:

(8) $$F_{D}=-12\pi \mu_{L}R\left(\overset{˙}{x}-v_{\mathrm{ex}}(x,t)\right)\text{.}$$

Hereby, $v_{\mathrm{ex}}(x,t)$ is the liquid velocity induced by the acoustic irradiation calculated at the centre of bubble.

### 2.3 Translation modelled by the time-averaged approach

By neglecting the mass of the bubble $m_{b}$, and integrating Eq. (4) for one acoustic period T=1/f, the averaged equation of translational motion is obtained as [92]

(9) $$\frac{\rho_{L}}{2}{\langle V\overset{¨}{x}\rangle }_{T}={\overset{‾}{F}}_{\mathrm{ex}}(x,t)\text{.}$$

where

(10) $$\langle \ldots \rangle_{T}≔1/T\int_{0}^{T}\ldots \;\mathrm{dt},$$

and ${\overset{‾}{F}}_{\mathrm{ex}}(x,t)$ is the time-averaged external force that is the sum of the time-averaged primary Bjerknes force and the drag force. These forces are calculated as

(11) $${\overset{‾}{F}}_{B1}=-{\langle \nabla p_{A}(\overset{¯}{x},t)\cdot V(t)\rangle }_{T},$$

(12) $${\overset{‾}{F}}_{D}=-12\pi \mu_{L}{\langle R_{n}(t)(\overset{˙}{\overset{¯}{x}}-v_{\mathrm{ex}}(x,t))\rangle }_{T},\;$$

The first term on the right hand side in Eq. (9) is the averaged-virtual mass term. By decoupling this term

(13) $${\overset{‾}{F}}_{M}=\frac{\rho_{L}}{2}{\langle V\overset{¨}{x}\rangle }_{T}\approx \frac{\rho_{L}}{2}{\langle V(t)\rangle }_{T}\overset{¨}{\overset{¯}{x}},$$

the equation of motion in averaged and the decoupled form is

(14) $${\overset{‾}{F}}_{M}={\overset{‾}{F}}_{B1}+{\overset{‾}{F}}_{D}\text{.}$$

The significant benefit of the decoupling of volumetric and translational motions is that once the average radial motion is known, it becomes straightforward to calculate the averaged forces. For its simplicity, it is the common technique to numerically simulate bubble clusters. It is worth to be mentioned, in most of the papers, the drag force is calculated according to an empirically obtained formula [50], [51], [95]; however, in the present paper, for the direct comparison of the numerical models, the averaged drag force is also calculated according to the Levich formula [94]; compare Eqs. (8), (12). It is worth mentioning that for the adequate treatment of the transient cycles, instead of pre-calculated tabulated forces as a function of bubble position [51], the averaging was carried out for each acoustic cycle simultaneously during the solution of the Keller–Miksis equation.

### 2.4 Properties of the standing wave field

The acoustic field is assumed to be a standing wave; thus, the far field pressure at the bubble position *x* is

(15) $$p_{\infty }(x,t)=P_{\infty }+p_{A}(x,t),$$

where $P_{\infty }=1\;\mathrm{bar}$ is the ambient pressure and $p_{A}(x,t)$ is the harmonic forcing. This term is written as

(16) $$p_{A}(x,t)=P_{A}\cdot \cos(\mathrm{kx})\cdot \sin(2\pi \mathrm{ft}),$$

where $P_{A}$ is the pressure amplitude, *f* is the driving frequency, k=2π/λ is the (angular) wavenumber and $\lambda =c_{L}/f$ is the wavelength. The derivative of the local pressure with respect the time is

(17) $${\overset{˙}{p}}_{A}(x,t)=P_{A}\cdot 2\pi f\cdot \cos(\mathrm{kx})\cdot \cos(2\pi \mathrm{ft}),$$

As the motion is restricted to one dimensional motion, the pressure gradient has only one component that is

(18) $$\nabla p_{A}(x,t)=-P_{A}\cdot k\cdot \sin(\mathrm{kx})\cdot \sin(2\pi \mathrm{ft}),$$

and the liquid velocity induced by the acoustic irradiation at the center of the bubble is

(19) $$v_{\mathrm{ex}}(x,t)=-\frac{p_{A}}{\rho_{L}c_{L}}\cdot \sin(\mathrm{kx})\cdot \cos(2\pi \mathrm{ft})\text{.}$$

## 3 Numerical technique

For numerical solution, the above equations were transformed into systems of ordinary differential equations by introducing dimensionless variables; namely, the dimensionless bubble radius $y_{1}=R/R_{0}$, the dimensionless position ξ=x/λ, the dimensionless time τ=t/(2π/ω) and the dimensionless bubble wall velocity $y_{1}^{\prime }=y_{2}$. The dimensionless system of equations in the case of time-resolved and time-averaged approaches are given in A.1, A.2, respectively. The dimensionless system of ordinary differential equations was solved by using an initial value problem solver.

For calculating simple time-series curves, the models are implemented in *Python* and solved by using the initial value problem solver called *solve*_*ivp* included in *SciPy* computational library. Depending on the stiffness of the problem, the built-in *‘RK45’* or *‘BDF’* methods were used. The high resolution, bi-parametric maps were calculated by using the MPGOS program packages [96], [97], [98]. MPGOS is an open-source, general-purpose program package, which is developed in C++ and CUDA C and capable to exploit the computing power of graphical processing units (GPUs). Its performance has already been demonstrated in [47], [99]. From the available initial value problem solvers, the *RKCK45* method was chosen, which is based on the fourth-order Runge–Kutta–Cash–Karp method with fifth-order error estimation. The high processing power of GPUs enables to carry out a considerable amount of numerical simulations within a reasonable time [100], [101], [102].

## 4 Quick overview of dynamical features, linear theory and model comparison

Depending on the bubble size, the excitation frequency and the driving amplitude the typical translational motion of the bubble can be classified [78]. The present section summarizes these different kinds of bubble motions and numerical examples are provided to compare the numerical models. In a weak standing wave, bubbles with size larger than the linear resonance translate to the pressure amplitude minima (node); on the contrary, bubbles with a size below the resonance size are pushed to the pressure maximum (antinode). At the antinodes, the pressure gradient is zero; thus, the primary Bjerknes force vanishes and the bubble position is in equilibrium. The node is also in an equilibrium position because of the zero pressure amplitude; therefore, the bubble is at rest and the time average of the Bjerknes force is zero. As the intensity of the acoustic irradiation is higher, the translational motion of the bubble is more complicated due to the nonlinear behaviour of the bubble. In this case, the large bubble volume expansion and the shift of the collapse phase relative to the driving pressure affects the absolute value and the sign of the Bjerknes force. Thus; the Bjerknes force may point away from the antinode even for small bubbles. In this case, stable positional equilibrium may exist between the antinode and node, where the acoustic pressure causes oscillation of small bubbles that lead to zero mean primary Bjerknes force. Besides stable equilibrium positions, the bubbles may demonstrate positional instability, for two reasons. In the first one, the bubble shape oscillation is parametrically excited by the volume oscillation. This causes the erratic small amplitude dancing motion of the bubble which was observed in [103], [104], [105], [106], [107]. In the present paper, this type of instability is not investigated as the models do not take into account the coupled shape mode oscillations [108], [109], [110], [111]. The second type of instability is caused by the change in the sign of the primary Bjerknes force close to the resonance bubble size, where hysteresis of the radial dynamics sets in. Hence, the bubble performs large amplitude oscillation in the space.

**Fig. 1:**
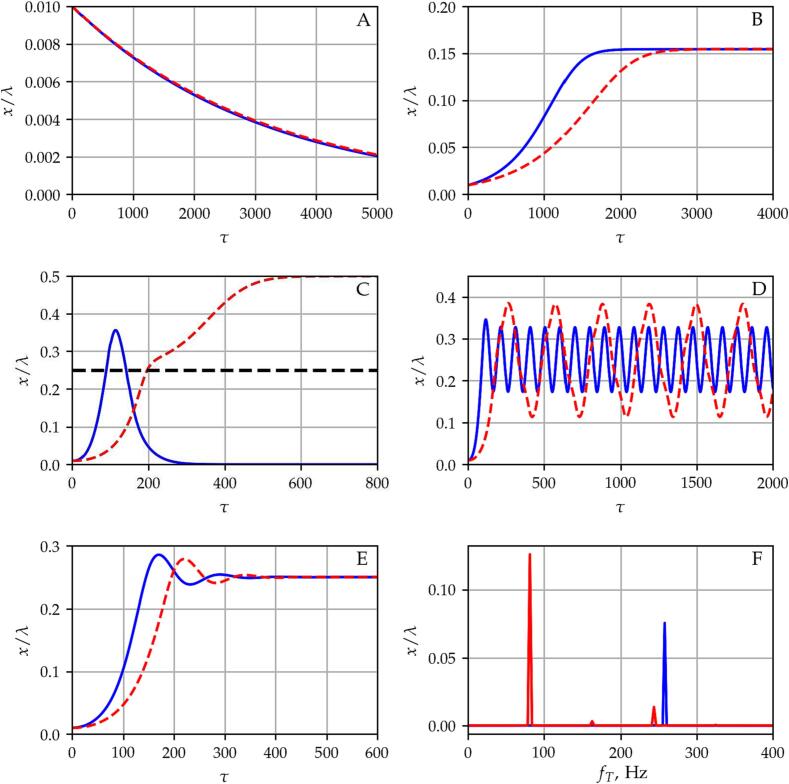
Panels A to E depict the bubble position in terms of relative displacement from the antinode as a function of the dimensionless time for bubble radii $R_{0}=20,\;60,\;100,\;110\;\mathrm{and}\;140\;\mu m$, respectively. Panel F shows the spectrum of the translational motion corresponding to the bubble path observed in the case of $R_{0}=120\;\mu m$ (Panel D). The dashed red and solid blue curves denote the numerical results obtained by means of the *Time-Averaged* and the *Time-Resolved* method, respectively. In panel C, the black dashed line denotes the plane of the first node.

Fig. 1 demonstrates the different kinds of the aforementioned translational motions obtained in a standing acoustic field. Panels A to E depict the bubble position in terms of the normalized distance from the pressure antinode (x=0) as a function of dimensionless time at bubble radii $R_{0}=20,\;60,\;100,\;110\;\mathrm{and}\;140\;\mu m$. In all the cases, the driving frequency and the pressure amplitude are constant, i.e., f=25kHz, and $p_{A}=60\;\mathrm{kPa}$. The solid blue and the dashed red curves denote the bubble path obtained by using the time-resolved and the time-averaged method, respectively.

The results presented on the left-hand side of Fig. 1 obey the linear theory [95]. The bubbles with a size below the resonance size of $R_{0,\mathrm{res}}=131.15\;\mu m$ corresponding to the excitation frequency f=25kHz, which is calculated from

(20) $$f_{0}=\frac{1}{2\pi }\sqrt{\frac{3\gamma (P_{\infty }-p_{V})}{\rho_{L}R_{0}^{2}}-\frac{2(3\gamma -1)\sigma }{\rho_{L}R_{0}^{3}}},$$

are attracted by the antinode (panels A and C), while in the case of an equilibrium bubble radius higher than the resonance size, the bubble is attracted by the node (panel E). Although the results obtained by the different models show qualitatively similar behaviour; the dynamics at $R_{0}=100\;\mu m$ (panel C) shows a significant difference. By using both the time-averaged and the time-resolved methods, the bubble is attracted by the antinodes (x/λ=0orx/λ=0.5). However, the bubble path obtained by the time-averaged method (dashed red curve) crosses the plane of the node (black dashed curve) and approaches to the next antinode. In panel B, the 60μm bubble has a stable equilibrium position near x/λ=0.155 between the corresponding antinode and node. In this case, the eigenfrequency of the bubble is $f_{0}=54.92\;\mathrm{kHz}$, which is close to the first harmonic resonance of the system; i.e., $f_{0}/f=54.92/25\approx 2.2$. In panel D, near the resonant size, the bubble shows translational oscillation around the node. Both the amplitude and the frequency of the translational oscillation are different, which are quantified by calculating the Fast Fourier Transform of the converged solutions. The calculated spectra are plotted in panel F, where $f_{T}$ denotes the frequency of the translational oscillations. The translational oscillation frequency of the bubble is $f_{T}=81.26\;\mathrm{Hz}$, and $f_{T}=257.87\;\mathrm{Hz}$ obtained by the time-averaged and time-resolved methods, respectively. This is more than a factor of three. In addition, the amplitude of oscillation obtained by the time-averaged model x/λ=0.126 is roughly 1.6 times higher than the amplitude of translational oscillation x/λ=0.076 provided by the time-resolved model.

From Fig. 1, one can observe that both models are capable to seek for equilibrium bubble positions; there are only small differences in the speed of convergence. The huge quantitative difference observed in the translational frequency implies that the time-resolved model is the proper choice in computations, where continuous translational dynamics plays an important role.

## 5 Model comparison in the parameter space of pressure amplitude, equilibrium radius and driving frequency

Investigating simple time-series curves is not feasible to conclude general observation; therefore, numerical investigations were carried out in a wide range of parameters. To ensure the convergence of the numerical solution, the first 25000 acoustic cycles were treated as the transient solution and discarded. The high number of acoustic periods is justified by the slow translational velocity compared to the radial oscillation, especially for week acoustic driving and small bubble radius as the Bjerknes force is proportional to $R^{3}$, see again Fig. 1A. The results obtained during the next 5000 acoustic cycles were treated as converged solutions and evaluated. In this case, the smoothed bubble trajectory, i.e., the bubble position and velocity at the end of each acoustic cycle was stored. These converged solutions were classified as stable and oscillating translational motion. The convergence to an equilibrium position was tested as the differences between the consecutive position and velocity values were less than pre-defined tolerances

(21) $$\left|\xi_{1,i}-\xi_{1,i+1}\right|<\varepsilon \;\mathrm{and}\;\left|\xi_{2,i}-\xi_{2,i+1}\right|<\varepsilon \;\mathrm{for}\;i\in [25\;000\text{;}\;30\;000]\text{.}$$

The tolerance was set as $\varepsilon =10^{-6}$. In the case of translational oscillation, the frequency and the amplitude of oscillation were calculated by the Fast Fourier Transform similar to Fig. 1.

To compare the capabilities of the numerical techniques on a wide range of parameters, bi-parametric simulations were carried out in the parameter plane of the equilibrium bubble size $R_{0}$ and pressure amplitude $p_{A}$ at fixed driving frequency values *f*. The investigated set of parameters and their resolutions are summarized in Table 1.

**Table 1:** The investigated parameter set, and the range of the applied initial conditions.

f,kHz	$R_{0,r},\mu m$	$R_{0}\;\mu m$	$p_{A},\;\mathrm{kPa}$	$N_{R_{0}}\times N_{p_{A}}$	x,mm	$N_{\mathrm{IC}}$
25	131.15	10–150	10–125	141×116	0.06–1.494	5
50	65.85	10–80	10–125	141×116	0.03–0.747	5
100	33.19	10–40	10–125	121×116	0.015–0.3735	5
200	16.85	5–20	10–150	151×141	0.0075–0.1867	5

The first column contains the excitation frequency and the second column contains the corresponding resonance radius $R_{0,r}$. The third and fourth columns show the parameter limits, and the applied resolution is depicted in the fifth column. At each parameter combination, initial value problem computations were carried out. The initial bubble size was prescribed as the equilibrium radius and the initial bubble wall velocity was set to zero. The velocity of the translational bubble motion was also set to zero. The initial bubble position was varied between the antinode and the next node. In terms of relative displacement, $N_{\mathrm{IC}}=5$ equally distributed initial bubble positions were prescribed between x/λ=0.01 and x/λ=0.249 to reveal the possible coexisting stable equilibrium positions. The applied limits of the initial displacement is given in the sixth column of Table 1. Thus; the total number of investigated parameter combinations is $5\times \left(141\times 116+141\times 116+121\times 116+151\times 141\right)=340\;195$.

The detailed comparisons of the models are presented in the following subsections. First, the results are processed in terms of stable equilibrium positions and their stability threshold. Here, only the equilibrium positions (Section 5.1) and the number of equilibrium positions (multi-stability, Section 5.2) are compared; that is, only the final state of the bubble is important. The stable equilibrium positions give no information about the transient behaviour of the bubble; thus, in the case of translating bubbles, the frequency and the amplitude of translational oscillation are compared in Section 5.3.

### 5.1 Stable positions

**Fig. 2:**
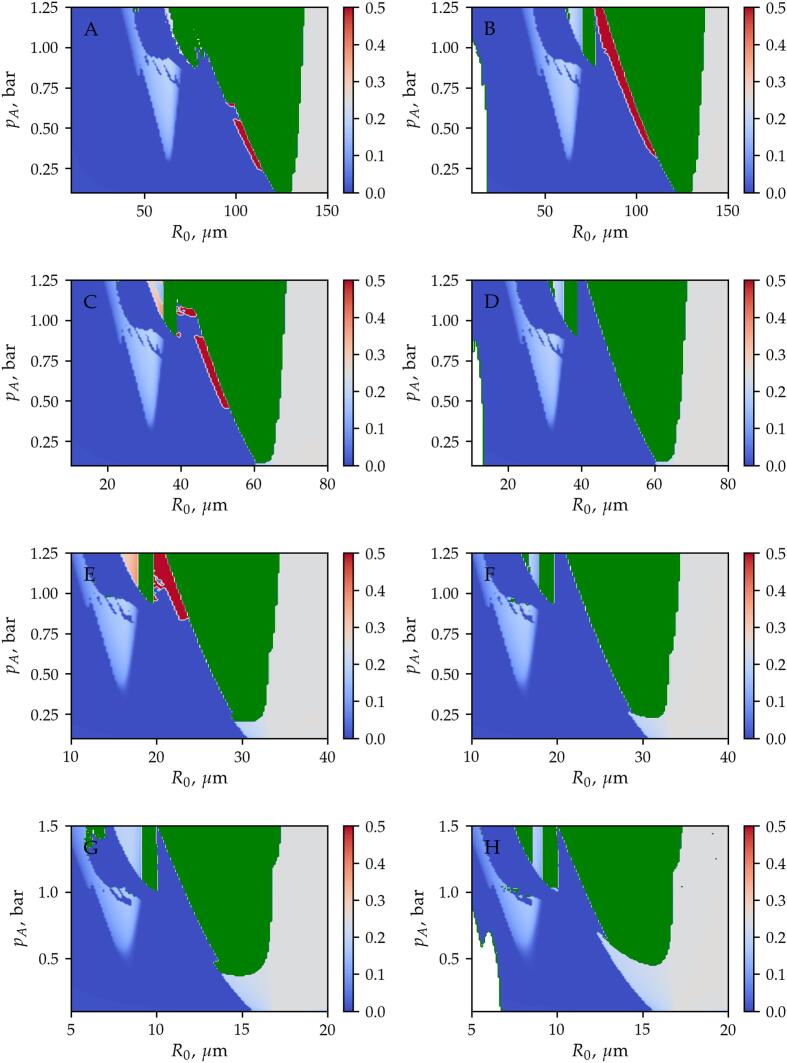
The stability maps obtained by means of the *Time-Resolved* (left column) and the *Time-Averaged* methods. The first, second, third and fourth rows correspond to excitation frequencies f=25,50,100,and200kHz, respectively. The colormap denotes the stable equilibrium position observed after the transient cycles. In addition, the green domain denotes translational oscillation, and the white domain appearing at low bubble radius $R_{0}$ and pressure amplitude $p_{A}$ (lower left corners of panels B, D and H) denotes the parameter range where the *Time-Averaged* method failed to run.

Fig. 2 shows the stable equilibrium positions obtained by the different methods as a function of the equilibrium radius $R_{0}$ and the pressure amplitude $p_{A}$. The first, second, third and fourth rows correspond to excitation frequencies f=25,50,100,and200kHz, respectively. The parameter maps on the left-hand side are obtained by the time-resolved approach, while the results on the right-hand side are calculated by means of the time-averaged method. For simplicity, only the equilibrium positions obtained by using x/λ=0.01 as the initial displacement of the bubble are presented in Fig. 2. The co-existing equilibrium positions revealed by using all the initial conditions are presented in the next subsection. The colourmap denotes the obtained stable equilibrium position in terms of the relative displacement (x/λ). The blue and red regions correspond to the antinodes located at x/λ=0 and x/λ=0.5, while the grey domain denotes the pressure minimum (node). In the green domain, the bubble exhibits translational oscillation discussed in details in Section 5.3.

The equilibrium positions obtained by the different methods show a good agreement and the gross features in terms of translational stability are captured by both numerical models. Above the resonance size, the bubble path converges to the closest node (grey domain) for each excitation frequency. Well beyond the resonance size, the bubble is attracted by the antinode even for higher pressure amplitude values. From the resonance bubble size, with increasing pressure amplitude, a large domain of translating bubble (green) emerges. Only slight differences in the stability map can be observed near the stability threshold (boundary of the green area), where the red stripes indicate convergence to a different antinode. Close to the first harmonic resonance, where the bubble size is half of its resonance size, an intermediate stable equilibrium position exists between the node and the antinode. The shape of the domains of intermediate equilibrium positions obtained by the different methods agree well. It is worth mentioning that near the harmonic resonances, multi-stability may exist due to the hysteresis and the appearance of higher periodic solutions in the radial dynamics of the bubble [112]. Multi-stability is discussed in the next subsection.

**Fig. 3:**
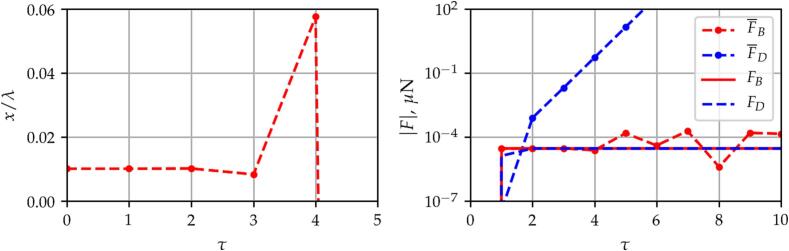
Example of numerical instability observed at small bubble size ($R_{0}=5\;\mu m$), for $p_{A}=0.5\;\mathrm{bar}$ pressure amplitude and f=25kHz excitation frequency. Left: the dimensionless bubble position as a function of the dimensionless time is depicted. Right: the forces exerted on the bubble as a function of the dimensionless time are plotted.

Note that there are narrow white regions in panels B, D and H in the lower left corners of the figures. In these regions, the time-averaged method is numerically ill-conditioned. The reason is the instability of the semi-implicit Euler method used to solve the decoupled translational motion. This instability of the numerical solution is demonstrated in Fig. 3. The figure is obtained for a bubble with an equilibrium radius of $R_{0}=5\;\mu m$ at $p_{A}=0.5\;\mathrm{bar}$ pressure amplitude and f=25kHz excitation frequency. On the left-hand side of the figure, the dimensionless displacement of the bubble is plotted as a function of the dimensionless time. The oscillation of the numerical solution is evident. The reason for this instability is the inaccuracy in the translational velocity provided by the Euler step. It is observable in the right-hand side of the figure where the forces exerted on the bubble are depicted as the function of the dimensionless time. The dotted-dashed lines correspond to the mean forces (${\overset{‾}{F}}_{B},{\overset{‾}{F}}_{D}$), while the solid and dashed lines denote the forces ($F_{B},F_{D}$) obtained by solving the same problem by means of the time-resolved method and then averaging the instantaneous forces. Note that the absolute values of the forces are plotted, and logarithmic scale is applied for the vertical axis. The drag force, which is proportional to the translational velocity increases rapidly with time. This induces a rapid increase in the net forces and results in numerical instability in the solution. The value of the drag force should maintain close to the values obtained by averaging the instantaneous forces obtained by the time-resolved method (solid and dashed lines). Note that the numerical instability may be resolved by using a higher-order numerical scheme to solve the decoupled equation of motion. As usually the semi-implicit Euler method is applied in the literature [50], [51], the present paper employed the same method and does not investigate the aforementioned numerical instability in detail. Although, such instability arises at low bubble sizes; it is limited to pressure amplitude values below the ambient pressure (1 bar), see Fig. 2 again. The typical sonochemical applications require pressure amplitude higher than the ambient pressure; thus, in the parameter range of real applications, the averaging method provides satisfactory results for a single bubble, i.e., capturing the attraction of the pressure antinode. In addition, the averaging method was used in particle simulations to capture key features of bubble clusters [50], [51], [113] and it has been validated for two bubbles experimentally [114].

### 5.2 Multi-stability

**Fig. 4:**
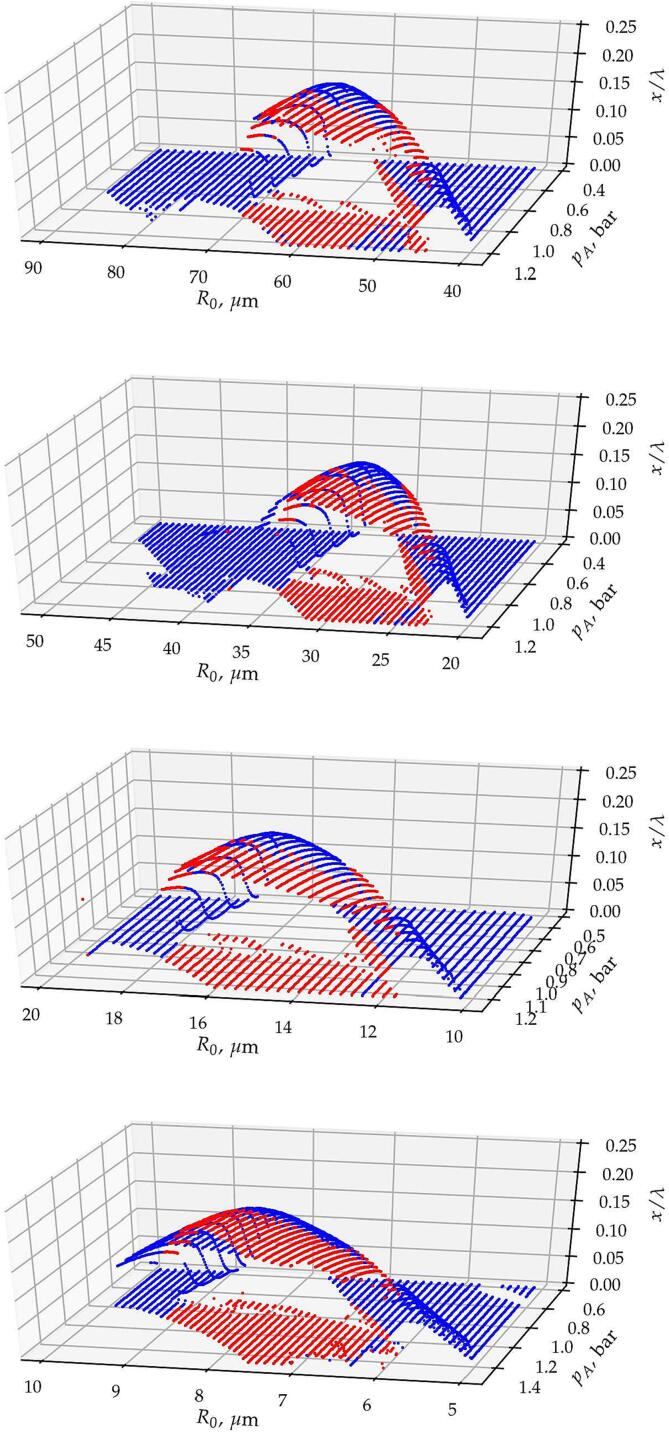
Equilibrium positions obtained by means of the time-resolved model as a function of the equilibrium radius and pressure amplitude.

To present the co-existence of multiple stable equilibrium positions, the non-oscillating solutions are plotted in 3D in Fig. 4. Keep in mind that the number of initial conditions at each set of parameters is five. In the diagrams, the stable bubble positions in terms the of relative displacement are plotted as a function of the equilibrium radii and pressure amplitude. The equilibrium positions found by both models are agreed very well; thus, for the clarity of the figures, the equilibrium positions obtained only by the time-resolved model are plotted on the figures. From the top to the bottom, the figures correspond to excitation frequencies 25,50,100 and 200kHz, respectively.

The figures show that intermediate equilibrium solutions exist near the harmonic resonance. These form a smooth surface in the parameter plane of pressure amplitude an equilibrium radius. There are co-existing equilibrium positions denoted by the red dots where this surface overlaps with the plane of equilibrium positions at x/λ=0.

### 5.3 Translating bubbles

**Fig. 5:**
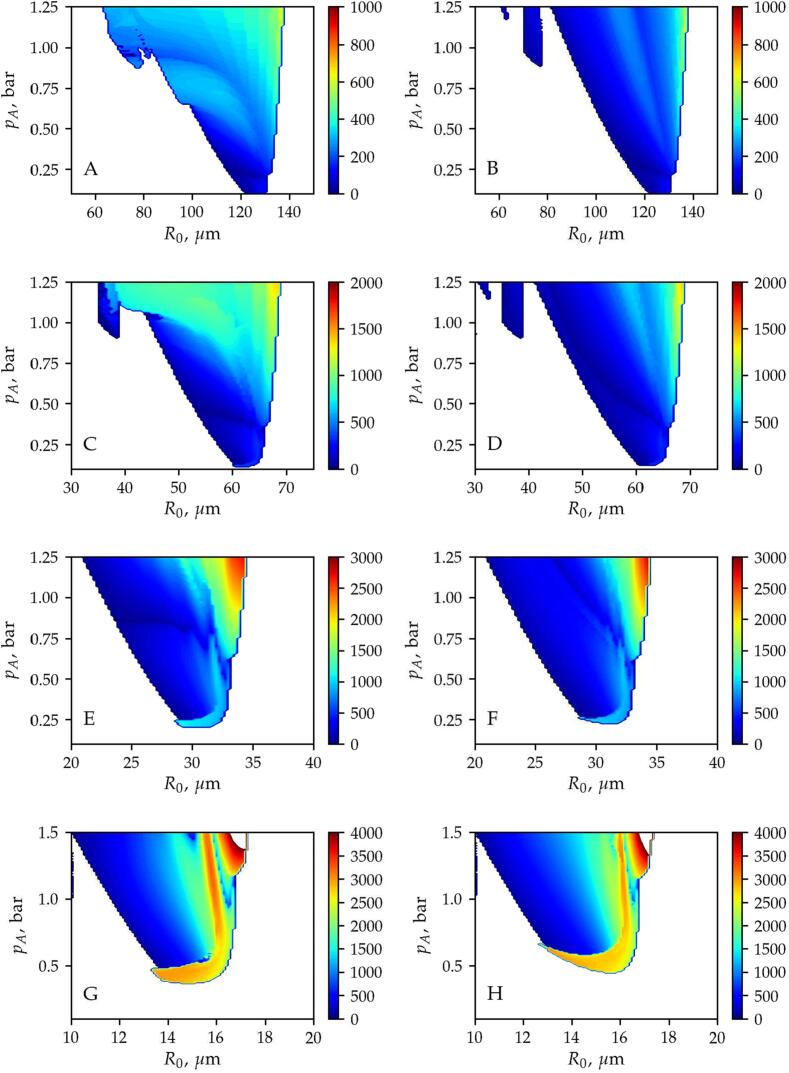
The oscillation frequency obtained by means of the *Time-Resolved* (left column) and the *Time-Averaged* methods for different excitation frequency values. The first, second, third and fourth rows correspond to excitation frequencies f=25,50,100,and200kHz, respectively. The colormap denotes the main oscillation frequency of the translational motion in Hz. The white domain denotes stable positions.

**Fig. 6:**
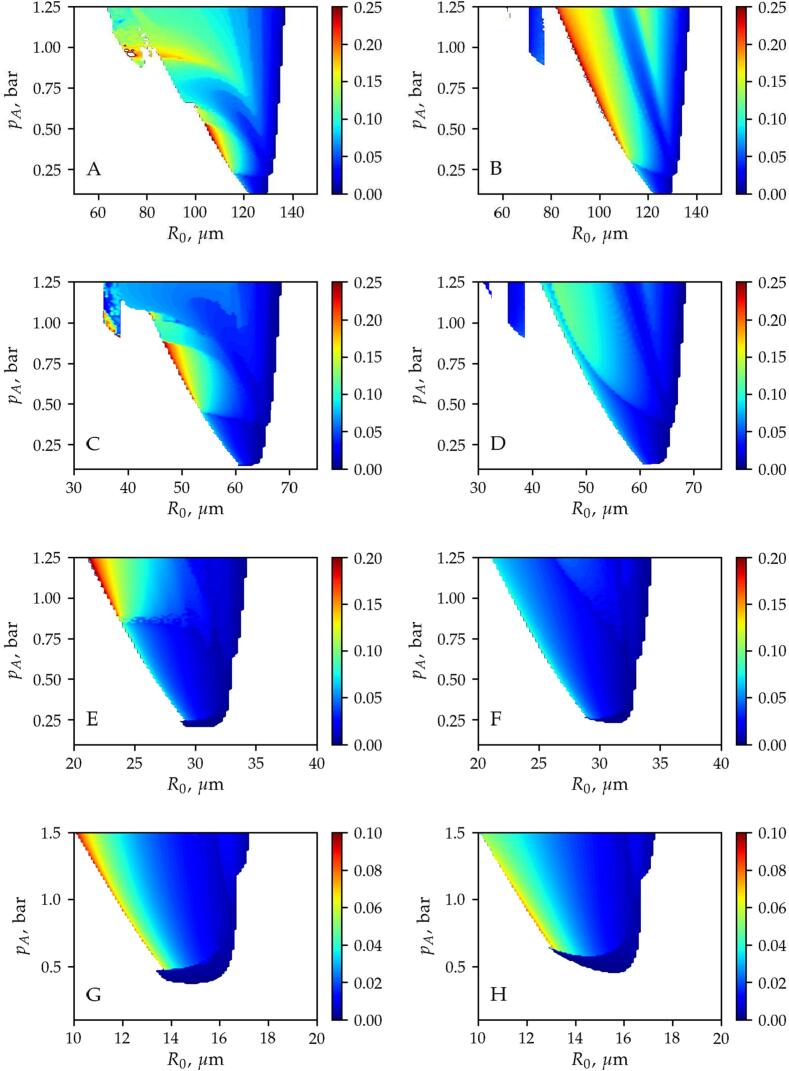
The oscillation amplitude obtained by means of the *Time-Resolved* (left column) and the *Time-Averaged* methods for different excitation frequency values. The first, second, third and fourth rows correspond to excitation frequencies f=25,50,100,and200kHz, respectively. The colormap denotes the main oscillation amplitude of the translational motion in terms of the relative displacement x/λ. The white domain denotes stable positions.

In the case of translating bubbles, the main oscillation frequency and the corresponding amplitude of the bubble motion obtained by FFT are plotted in Fig. 5, Fig. 6, respectively. Note that the colourmap for the oscillation frequency of the translational motion shows the main frequency in Hz (Fig. 5), while the oscillation amplitude (Fig. 6) is plotted in terms of relative displacement (x/λ). Again, the first, second, third and fourth rows correspond to excitation frequencies f=25,50,100,and200kHz, respectively. In addition, the figures on the left-hand side are obtained by the time-resolved approach and the figures on the right-hand side are calculated by the time-averaged method.

The side-by-side comparison of the figures shows that the oscillation frequency of the translational motion is 2–3 times higher for pressure amplitude above $p_{A}=0.5\;\mathrm{bar}$ in the case of f=25kHz and f=50kHz excitation frequencies (first and second rows). As the excitation frequency is increased, the difference in the frequency of the translational motion is getting smaller, see Fig. 5. Also with increasing excitation frequency the amplitude of translational motion (Fig. 6) decreases for both methods and the results are getting closer. To sum up, for translating bubbles, the application of the time-resolved method is mandatory to capture correctly the bubble path for excitation frequencies below f=50kHz.

**Fig. 7:**
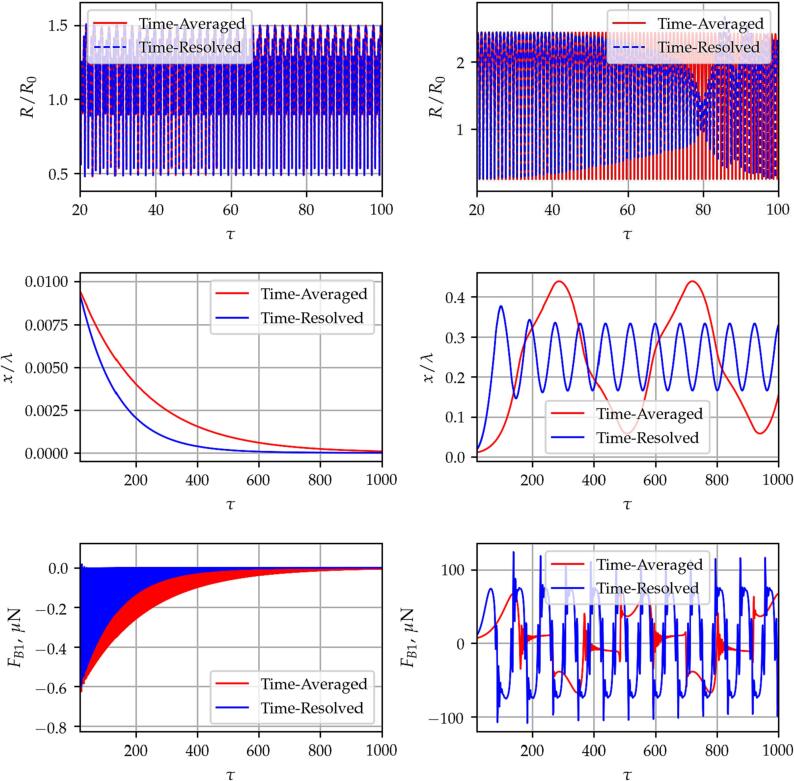
The dimensionless bubble radius $R/R_{0}$, the relative bubble displacement x/λ and the averaged Bjerknes force $F_{B1}$ as a function of the dimensionless time obtained by using the *Time-Averaged* (red) and the *Time-Resolved* (blue) methods. The excitation frequency is f=25kHz and the pressure amplitude is $p_{A}=0.75\;\mathrm{bar}$. The graphs on the left- and right-hand sides are calculated for equilibrium radii $R_{0}=80\;\mu m$ and $R_{0}=100\;\mu m$, respectively.

An example of the difference in the oscillating solution is presented in Fig. 7, where the dimensionless bubble radius $R/R_{0}$, the relative displacement x/λ and the average Bjerknes force $F_{B1}$ are plotted as a function of the dimensionless time. The red and blue curves denote the results obtained by the time-averaged and the time-resolve methods, respectively. Note that in the case of the time-resolved method, the instantaneous forces were calculated during the integration, and then the results were averaged for each acoustic cycle. The results on the left- and right-hand sides are obtained for bubble radii $R_{0}=80\;\mu m$ (below resonance) and $R_{0}=100\;\mu m$ (near the resonance), respectively. The excitation frequency is f=25kHz and the pressure amplitude is $p_{A}=0.75\;\mathrm{bar}$.

By comparing the diagrams, one can see that below the resonance size of the bubble, the radius-time curves show a relatively good agreement. The primary Bjerknes force is a negative number; thus, the bubble approaches the closest antinode at x/λ=0. Only a small difference in the speed of convergence can be observed. This is in accordance with the results presented in Section 5.1 and in Fig. 2. Near the resonance; however, the fluctuating pressure amplitude (due to the alteration of the bubble position) induces complicated bubble oscillation and translational motion [78]. Such behaviour are not captured by the time-averaged models; thus, the radius-time curves and the Bjerknes-force time curves show significant differences.

## 6 Summary and discussion

The translational motion of cavitation bubbles is responsible for various forms of bubble clusters. Depending on the acoustic field (standing wave, travelling wave) different distributions of bubbles have been observed experimentally, such as the cone at the top of the transducer [113], or streamers [50], [51], [52], [54]. The common property of the observed clusters is that the bubbles do not fill the liquid container evenly; thus, it lets a massive amount of passive zones in a sonochemical reactor. These zones results in non-uniform cavitational activity, which is one of the main barriers to the scalability of sonochemical applications. Another limitation is the attenuation of the sound waves (acoustic shielding [48]) due to the densely packed bubble clusters near the irradiation surface, such as at the top of an immersed transducer. Due to the high energy dissipation near the irradiating surface, the penetration depth of the irradiation is approximately a few centimetres [49].

The simple idea of increasing the irradiation intensity to face the scalability of sonochemical reactors fails due to the aforementioned reasons. As the intensity of the irradiation is proportional to the square of the pressure amplitude ($I\propto p_{A}^{2}$); a small increase in the pressure amplitude decreases the energy efficiency of the reactor. The high pressure amplitude induces strong collapse; however, there is an optimal range of bubble collapse strength, which depends on the application [3]. In addition, the higher the pressure amplitude the more the number of the generated bubbles, and the resulting higher density of bubbles enhances the acoustic shielding effect as well. The possible solution is to optimize a sonochemical reactor by manipulating the acoustic field to achieve homogeneous bubble distribution. As the number of involved parameters is high, and the dynamics of a bubble show nonlinear behaviour, the numerical simulations are important tools in the field of sonochemistry. In a high dimensional parameters space by applying a moderate resolution for each parameter, the number of parameter combinations can be extremely high. An example for the investigation of a single bubble in dual frequency covered nearly 2 billion parameter combinations [47]. Therefore; to develop reliable control techniques, an efficient but reliable numerical model is required. The accurate models can be widely used to properly describe bubble dynamics in applications where translational motion is essential. Besides the sonochemical applications, translational motion is important in medical applications as well. In the case of clot lysis [115], [116], the ultrasound-stimulated microbubbles are pushed close to the clot.

In general, the modelling of the translating and oscillating bubble requires solving the governing equations describing the radial oscillation and the equation of motion for the translation. There are two main approaches exits in the literature. The trivial technique is to solve the system of the coupled ordinary differential equation describing the translational motion and radial oscillations, which is referred to as the Time-Resolved method in the present paper. The second approach is to decouple the radial oscillation and the translational motion. In this case, the forces acting on the bubble are averaged for one acoustic cycle and the translation of motion is solved by using the averaged values. Although, studies have already been published investigating the validity of the decoupling approximation [91], [92], they are *limited to a few parameter combinations or weak acoustic forcing*. The present study compares the results provided by both modelling approaches on a wider range of parameter combinations, which is not limited to small amplitude oscillation. Therefore, qualitatively different dynamics were identified in the oscillatory regime.

The results showed that in terms of equilibrium positions, both models give the same results. Below the resonance size, the bubble is attracted by the antinode, and above the resonance size, the bubble tends to approach to the node. This behaviour of the bubble is well known for weak standing acoustic fields, which is described by the linear theory [81]. In addition, it was observed that close to the higher harmonic resonances, intermediate equilibrium positions between the node and the antinode may exist. Contrary to the good agreement in terms of translational equilibrium, there are significant differences for continuously translating bubbles. In this case, the bubble oscillates if the bubble size is close to the resonance size. It must be emphasized that the models contains simplifications. In the case of the low frequency and high amplitude region (Giant Response region [44]), the bubble wall velocity calculated by the Keller–Miksis equation may exceed the speed of sound in the liquid. Yasui [117] proved that based on the fundamental theory of fluid dynamics, the bubble wall Mach number never exceeds M=1; thus, in this case, the correction of the model describing the radial dynamics would be necessary [118]. In addition, the equation of motion describing the translational motion neglect the effect of the complex flow around the bubble. Corrections are available to take into account the effect of the drag force acting on the bubble by adding the history force depending on the value of the translational Reynolds number Ret=R|u|/ν and the radial Reynolds number Rer=R|R˙|ν [119], [120], [121]. Reddy and Szeri [91] proved that for violent bubble collapse, when Ret>1 or Rer>1, the history force is negligible. Further improvement in the model accuracy can be achieved by including the history force.

In the case of a single bubble, the complexity of both models are quite simple. However, for bubble clusters containing thousands of bubbles, the interaction of individual bubbles via their emitted pressures (e.g., via the secondary Bjerknes force [122], [113], [50], [114], [51]) has to be taken into account. The secondary Bjerknes force can be attractive or repulsive for a pair of bubbles depending on the equilibrium radii and the properties of the acoustic field that complicates the prediction of the cluster behaviour. The calculation of the secondary Bjerknes force for bubble clusters by using the time-averaged approach includes simplifications such as equally varying bubble volume; thus, the complexity of the model does not increase significantly. On the contrary, in the case of the time-resolved model, the differential equations include the coupling terms up a given orders [80], [89], [88] in terms of inverse separation distance. These equations contain implicit terms that emerge numerical difficulties, i.e., at every timestep the evaluation of the right-hand side of the ODE requires solving a system of linear equations, which makes the efficient implementation of the models challenging (for example in CUDA to exploit the processing of power of GPUs). An important advantage of solving the full ODE instead of averaging is to take into account the time delay caused by the compressibility of the liquid in the bubble–bubble interaction [123], [124]. The time delay may have a huge influence on the radial dynamics and thereby the translational motion as well, especially in the transient regimes. The explicit inclusion of the time delay makes the simulations computationally intensive as the delay differential equations have large memory requirement, which limits the size of the bubble cluster in simulations [83]. A promising approach is to decompose a model into subclusters and handle the interaction between subclusters [125]. The present results obtained in the case of a single bubble imply that the precise prediction of bubble-cluster behaviour requires the solution of the time-resolved approach [90], in spite of the arising numerical difficulties.

## CRediT authorship contribution statement

**Kálmán Klapcsik:** Conceptualization, Data curation, Formal analysis, Funding acquisition, Investigation, Methodology, Software, Validation, Visualization, Writing – original draft, Writing – review & editing. **Ferenc Hegedűs:** Project administration, Funding acquisition, Supervision, Software, Writing – original draft, Writing – review & editing.

## Declaration of Competing Interest

The authors declare that they have no known competing financial interests or personal relationships that could have appeared to influence the work reported in this paper.

## Appendix A Dimensionless system of equations

### A.1 Time resolved method

The mathematical models described in Section 2 are rewritten into a dimensionless system of differential equations by introducing dimensionless variables; namely, the dimensionless bubble radius $y_{1}=R/R_{0}$; the dimensionless bubble wall velocity $y_{1}^{\prime }=y_{2}$, the dimensionless position $\xi_{1}=x/\lambda$ and the dimensionless translation velocity $\xi_{2}=\xi_{1}^{\prime }$, where the prime denotes the derivative with respect to the dimensionless time τ=t/(2π/ω). The dimensionless system in the case of the time-resolved approach is written as

(A.1) $$y_{1}^{\prime }=y_{2},$$

(A.2) $$y_{2}^{\prime }=\frac{N}{D},$$

(A.3) $$\xi_{1}^{\prime }=\xi_{2},$$

(A.4) $$\xi_{2}^{\prime }=\frac{3F_{\mathrm{ex}}\cdot C_{10}}{x_{1}^{3}}-\frac{3y_{2}\xi_{2}}{y_{1}},$$

where *N* and *D* are

(A.5) $$N=\left(C_{0}+C_{1}\cdot y_{2}\right){\left(\frac{1}{y_{1}}\right)}^{C_{8}}-C_{2}\left(1+C_{7}y_{2}\right)-C_{3}\frac{1}{y_{1}}-C_{4}\frac{y_{2}}{y_{1}}-\frac{3}{2}y_{2}^{2}\left(1-C_{7}\frac{y_{2}}{3}\right)-\left(1+C_{7}y_{2}\right)C_{5}\cdot p_{A}-C_{6}\cdot {\overset{˙}{p}}_{A}y_{1}+\frac{1}{4}\xi_{2}^{2}C_{9},$$

(A.6) $$D=y_{1}-C_{7}y_{1}y_{2}+C_{4}C_{7}\text{.}$$

The local pressure $p_{A}$ and its time derivative ${\overset{˙}{p}}_{A}$ are the functions of the dimensionless variables

(A.7) $$p_{A}=P_{A}\cdot \cos(2\pi \xi_{1})\cdot \sin(2\pi \tau ),$$

(A.8) $${\overset{˙}{p}}_{A}=P_{A}\omega \cos(2\pi \xi_{1})\cdot \cos(2\pi \tau )\text{.}$$

The external force is the sum of the instantaneous primary Bjerknes force $F_{B1}$ and the drag force $F_{D}$. These forces are calculated as

(A.9) $$F_{B1}=-C_{11}y_{1}^{3}\cdot \nabla p_{A},$$

(A.10) $$F_{D}=-C_{12}y_{1}\cdot \left(\xi_{2}c_{L}-v_{\mathrm{ex}}\right),$$

where $\nabla p_{A}$ is the pressure gradient

(A.11) $$\nabla p_{A}=-P_{A}\frac{2\pi }{\lambda }\cdot \sin(2\pi \xi_{1})\cdot \sin(2\pi \tau ),$$

and $v_{\mathrm{ex}}$ is the liquid velocity induced by the acoustic field

(A.12) $$v_{\mathrm{ex}}=-\frac{P_{A}}{\rho_{L}c_{L}}\cdot \sin(2\pi \xi_{1})\cdot \cos(2\pi \tau )\text{.}$$

Note that in the above equations $C_{0}-C_{24}$ are precomputed constants, which are independent of the time or position of the bubble. These constants are summarized in A.3.

### A.2 Time averaged method

The time averaged forces were calculated by changing the variable

(A.13) $${\langle \ldots \rangle }_{T}=\frac{1}{T}\int_{0}^{T}\ldots \;\mathrm{dt}=\frac{\omega }{2\pi }\int_{0}^{1}\ldots \;\frac{2\pi }{\omega }d\tau =\int_{0}^{1}\ldots d\tau ={\langle \ldots \rangle }_{1}\text{.}$$

By using the above notation, the averaged forces are calculated as

(A.14) $${\overset{‾}{F}}_{B1}=-C_{11}{\langle y_{1}^{3}\nabla p_{A}\rangle }_{1},$$

(A.15) $${\overset{‾}{F}}_{D}=-C_{12}\cdot {\langle y_{1}\left({\overset{¯}{\xi }}_{2}\cdot c_{L}-v_{\mathrm{ex}}\right)\rangle }_{1}\text{.}$$

The averaged mass force is the sum of the averaged primary Bjerknes and the averaged drag force. Then, according to Eq. (A.13), the averaged bubble translation acceleration is expressed as

(A.16) $${\overset{¯}{\xi }}_{2}^{\prime }=3\frac{{\overset{‾}{F}}_{M}C_{10}}{{\langle y_{1}^{3}\rangle }_{1}}\text{.}$$

The bubble position and velocity is updated according to the semi-implicit Euler method. Note that the timestep of the translational motion is one acoustic cycle which is one unit in the case of the dimensionless system; thus, the translational motion is updated as

(A.17) $${\overset{¯}{\xi }}_{2,j}={\overset{¯}{\xi }}_{2,j-1}+{\overset{¯}{\xi }}_{2,j}^{\prime },$$

(A.18) $${\overset{¯}{\xi }}_{1,j}={\overset{¯}{\xi }}_{1,j-1}+{\overset{¯}{\xi }}_{2,j},$$

where *j* counts the number of acoustic cycles.

The integral terms appearing in the above equations are calculated numerically by integrating the functions simultaneously with the dimensionless Keller–Miksis equation. Therefore; the following set of dimensionless ordinary differential equations are solved for each acoustic cycle

(A.19) $$y_{1}^{\prime }=y_{2},$$

(A.20) $$y_{2}^{\prime }=\frac{N}{D},$$

(A.21) $$f_{1}^{\prime }=y_{1}^{3},$$

(A.22) $$f_{2}^{\prime }=y_{1}\left({\overset{‾}{\xi }}_{2}\cdot c_{L}-v_{\mathrm{ex}}\right),$$

(A.23) $$f_{3}^{\prime }=\nabla p_{A}\cdot y_{1}^{3}\text{.}$$

Note that for a given acoustic cycle the bubble position and the bubble translational velocity were constant. The variables; such as $N,D,\nabla p_{A}$ and $v_{\mathrm{ex}}$ were calculated according to the formulas described in the previous section, except *N*. In the case of *N* the feedback term from the translational motion ($\xi_{2}^{2}C_{9}/4$) was neglected.

### A.3 Constant values

(A.24) $$C_{0}=\frac{1}{\rho_{L}}\left(\frac{2\sigma }{R_{0}}+P_{\infty }-p_{v}\right){\left(\frac{2\pi }{R_{0}\omega }\right)}^{2},$$

(A.25) $$C_{1}=\frac{1-3\gamma }{\rho_{L}c_{L}}\left(\frac{2\sigma }{R_{0}}+P_{\infty }-p_{v}\right)\frac{2\pi }{R_{0}\omega },$$

(A.26) $$C_{2}=\frac{P_{\infty }-p_{v}}{\rho_{L}}{\left(\frac{2\pi }{R_{0}\omega }\right)}^{2},$$

(A.27) $$C_{3}=\frac{2\sigma }{\rho_{L}R_{0}}{\left(\frac{2\pi }{R_{0}\omega }\right)}^{2},$$

(A.28) $$C_{4}=\frac{4\mu_{L}}{\rho_{L}R_{0}^{2}}\frac{2\pi }{\omega },$$

(A.29) $$C_{5}=\frac{1}{\rho_{L}}{\left(\frac{2\pi }{R_{0}\omega }\right)}^{2},$$

(A.30) $$C_{6}=\frac{R_{0}}{\rho_{L}c_{L}}{\left(\frac{2\pi }{R_{0}\omega }\right)}^{2},$$

(A.31) $$C_{7}=\frac{R_{0}\omega }{2\pi c_{L}},$$

(A.32) $$C_{8}=3\gamma ,$$

(A.33) $$C_{9}={\left(\frac{\lambda }{R_{0}}\right)}^{2},$$

(A.34) $$C_{10}=\frac{2\pi }{\rho_{L}R_{0}\lambda {\left(R_{0}\omega \right)}^{2}},$$

(A.35) $$C_{11}=\frac{4}{3}\pi R_{0}^{3},$$

(A.36) $$C_{12}=12\pi \mu_{L}R_{0}\text{.}$$
